# Differential effects of early life adversity on male and female rhesus macaque lifespan

**DOI:** 10.1002/ece3.10689

**Published:** 2023-11-05

**Authors:** Stephanie J. Gonzalez, Anthony J. Sherer, Raisa Hernández‐Pacheco

**Affiliations:** ^1^ Department of Biological Sciences California State University Long Beach California USA; ^2^ Department of Ecosystem Science and Management Pennsylvania State University University Park Pennsylvania USA

**Keywords:** Cayo Santiago, early life adversity, life history evolution, rhesus macaques, survival analysis

## Abstract

Early life adversity predicts shorter adult lifespan in several animal taxa. Yet, work on long‐lived primate populations suggests the evolution of mechanisms that contribute to resiliency and long lives despite early life insults. Here, we tested associations between individual and cumulative early life adversity and lifespan on rhesus macaques at the Cayo Santiago Biological Field Station using 50 years of demographic data. We performed sex‐specific survival analyses at different life stages to contrast short‐term effects of adversity (i.e., infant survival) with long‐term effects (i.e., adult survival). Female infants showed vulnerability to multiple adversities at birth, but affected females who survived to adulthood experienced a reduced risk later in life. In contrast, male infants showed vulnerability to a lower number of adversities at birth, but those who survived to adulthood were negatively affected by both early life individual and cumulative adversity. Our study shows profound immediate effects of insults  on female infant cohorts and suggests that affected female adults are more robust. In contrast, adult males who experienced harsh conditions early in life showed an increased mortality risk at older ages as expected from hypotheses within the life course perspective. Our analysis suggests sex‐specific selection pressures on life histories and highlights the need for studies addressing the effects of early life adversity across multiple life stages.

## Graphical abstract

Recent work on long‐lived primate populations suggests the evolution of mechanisms that contribute to resiliency and long lives despite early life insults. Our study shows profound immediate effects of insults at birth on female infant cohorts and suggests that affected female adults are more robust. In contrast, adult males who experienced harsh conditions early in life showed an increased mortality risk at older ages as expected from hypotheses within the life course perspective.

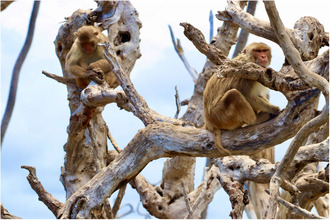

## 1 INTRODUCTION

Adversity early in life is hypothesized to reduce fitness components and thus influence the evolution of populations (Gluckman et al., 2008; Lindström, 1999). Emerging evidence now spans across several animal taxa (Snyder‐Mackler et al., 2020) and includes associations between early life adversity and mortality in adult mammals (e.g., low social rank, maternal death; Gicquel et al., 2022; Tung et al., 2016) and birds (e.g., low temperature, high density; Berntsen & Bech, 2016; De Kogel, 1997), and associations between early life adversity and health‐related stress responses later in life (e.g., glucocorticoids level; Grace & Anderson, 2018; Patterson et al., 2021; Rosenbaum et al., 2020). Multiple sources of early life adversity are also associated with the pace of reproduction (Belsky et al., 1991; Rickard et al., 2014) and consequent lifetime reproductive success (Descamps et al., 2008; Gicquel et al., 2022; Mumby et al., 2015; Pigeon & Pelletier, 2018; Sloboda et al., 2009). Yet, recent work on long‐lived primate populations questioned the role of the early life environment on fitness components. Primate populations may evolve protective mechanisms that contribute to resiliency and long lives despite adverse conditions early in life (Morrison et al., 2023). If primate populations have multiple reproductive events, then they may also evolve life history strategies to optimize reproductive schedules and compensate for shorter lifespans (Luevano et al., 2022; Weibel et al., 2020). Lastly, primate cohorts exposed to insults early in life may show significantly greater longevity due to viability selection during juvenile years (i.e., high‐quality juveniles who survive insults; Morrison et al., 2023).

Here, we aim to further our understanding of how the early life environment affects the lifespan of a long‐lived primate population. Exposure to individual insults early in life may affect a population through increased mortality during vulnerable stages (e.g., immature stages; Rosa et al., 2014; Zipple et al., 2020). If significant, such mortality may have profound effects on cohorts as it not only reduces population abundance but can also alter the distribution of phenotypes in the breeding population (e.g., age at maturity; Gosselin & Qian, 1997; Martin et al., 2018; Promislow & Harvey, 1990; Stearns & Koella, 1986). For those individuals surviving to adulthood, early life adversity may have far‐reaching consequences for health and survival by promoting disease and accelerated aging through stress‐induced biological mechanisms (Patterson et al., 2023; Polsky et al., 2022) and physiological changes such as inflammation and disease risk (Kinnally et al., 2019; Nettle et al., 2017). Given these multiple ways in which early life adversity can influence eco‐evolutionary process within populations, it is important to quantify and contrast its effects across different life stages.

Despite evidence of the role that early life adversity has on lifespan, understanding the link between adverse conditions early in life and survival in uncontrolled natural scenarios remains challenging. In such scenarios, it is often not possible to disentangle underlaying mechanisms driving individual responses (e.g., resiliency, trade‐offs). Data on social mammals are further limited to the non‐dispersing sex. Studies on primate populations with social dispersal suffer from the limitation of being biased toward a single sex, which often results in hypotheses being tested only in females (Campos et al., 2020). Understanding the influence of the early life environment on male longevity is crucial to enhance our knowledge of evolutionary processes. If the male response to early life insults differs from that of females, sex‐specific selection pressures on life histories are expected. However, if responses are similar, the contribution of males to eco‐evolutionary processes within populations might have previously been understated (Campos et al., 2020).

In this study, we extend previous work on the effects of early life adversity on the lifespan of a long‐lived primate population who showed that ecological sources of adversity early in life influence female reproduction (Luevano et al., 2022). We focus on multiple individual and cumulative sources of adversity on the rhesus macaque population at the Cayo Santiago Biological Field Station and carry out sex‐specific survival analyses at different life stages to contrast the short‐term effects of adversities (i.e., infant survival) with long‐term effects (i.e., adult survival). Given that male dispersal is constrained to the island of Cayo Santiago, this population is ideal for testing sex‐specific responses to early life adversity. Using 50 years of data, we first evaluate the short‐term effects of adversities by testing associations between the environment experienced at birth and infant survival. We predict that short‐term effects are negative and stronger, relative to long‐term effects, given the high vulnerability of infants to insults in altricial species (French & Carp, 2016; Tottenham, 2012; Zhang, 2017). Second, we evaluate the long‐term effects of adversities for those individuals surviving to adulthood by testing associations between the environment experienced from birth to juvenility (i.e., early life period) and adult survival. We predict a significant but weaker negative association between early life adversity and adult survival, relative to short‐term effects, likely due to viability selection (Morrison et al., 2023). Finally, we predict that the accumulation of adversities will have a stronger negative effect on both short‐ and long‐term survival, relative to individual adversity effects, because adverse conditions early in life may act in aggregate to influence adult health and survival (Hatch, 2005; O'Rand, 1996).

## 2 METHODS

### 2.1 Study population

**FIGURE 1:**
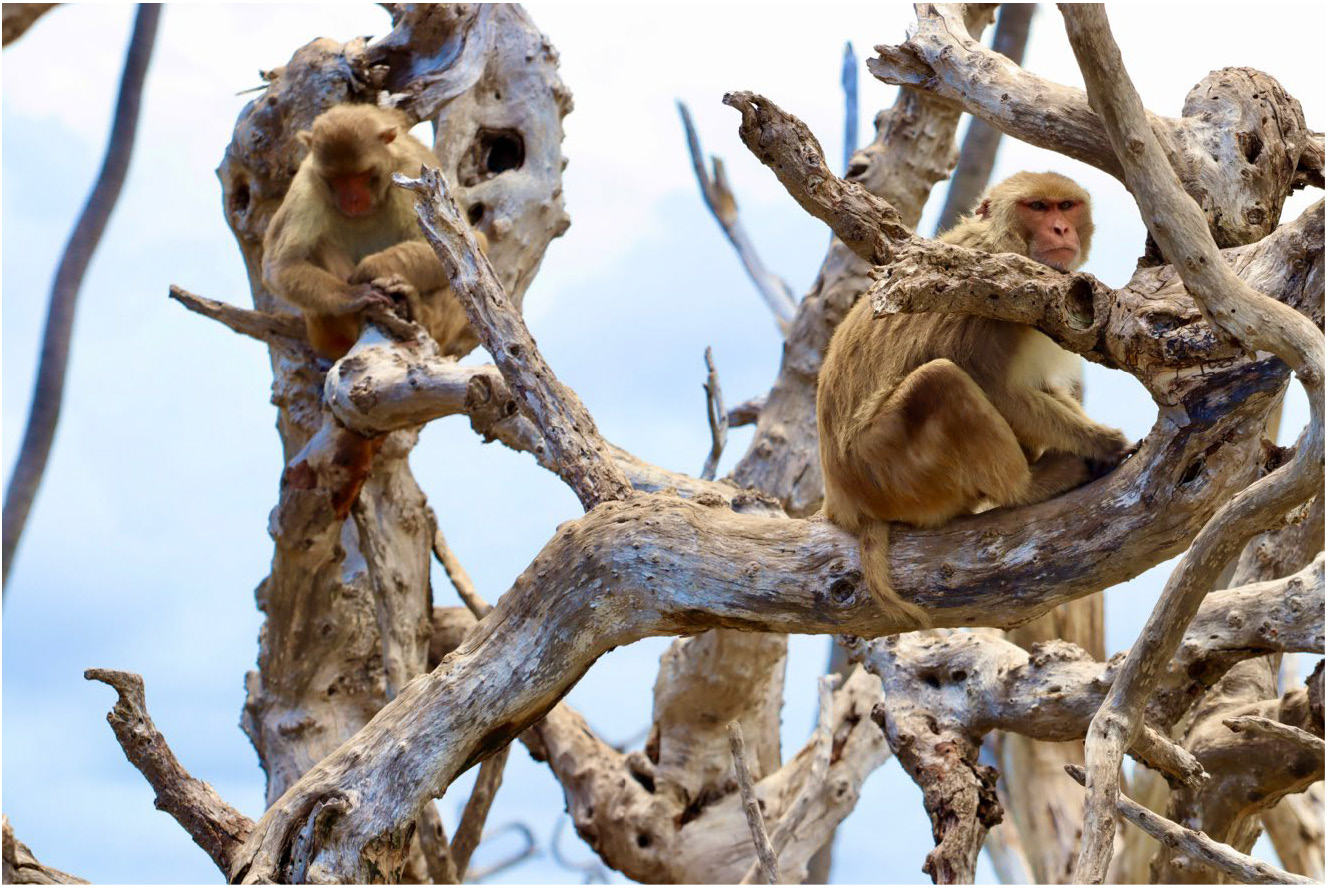
Two adult male rhesus macaques (*Macaca mulatta*) resting on a dead tree in Cayo Santiago following major hurricane Maria which impacted the island on September 20, 2017.

Cayo Santiago (CS) is a 15.2 ha island located 1 km off the southeastern coast of Puerto Rico (lat. 18°09′ N, long. 65°44′ W) that serves as biological field station for behavioral and noninvasive research of free‐ranging rhesus macaques (*Macaca mulatta*; Figure 1). The field station was established in 1938 from 409 Indian‐born monkeys being released onto the island, and no new individuals have been introduced since (Kessler & Rawlins, 2016). The population is kept under naturalistic conditions allowing the natural occurrence of synchronized annual birth seasons, social groups, and social dispersal (Hernández‐Pacheco, Rawlins, et al., 2016; Ruiz‐Lambides et al., 2017). These rhesus macaques forage on natural vegetation, have ad libitum access to water, and ad libitum high‐protein monkey chow distributed daily at approximately 0.23 kg/animal/day. Offspring start feeding on solid food in the first few months of life and are usually weaned by the end of their first year of life (Maestripieri & Hoffman, 2012). Veterinary intervention is restricted to the annual trapping season when yearlings are captured, marked for identification via ear notch and a unique three‐character tattoo, tetanus vaccines are administered to 1‐ and 2‐year‐old subjects, and physical samples are collected. During the trapping season, some individuals of different ages and sex may be permanently removed from the island to control for population size (Hernández‐Pacheco, Delgado, et al., 2016). Regular visual censuses report on the date of birth, sex, maternal genealogy, social group membership, and date of death or permanent removal from the island for every individual in the population immediately or within 2 days of occurrence (Ruiz‐Lambides et al., 2017).

### 2.2 Adversity at birth and infant survival

**FIGURE 2:**
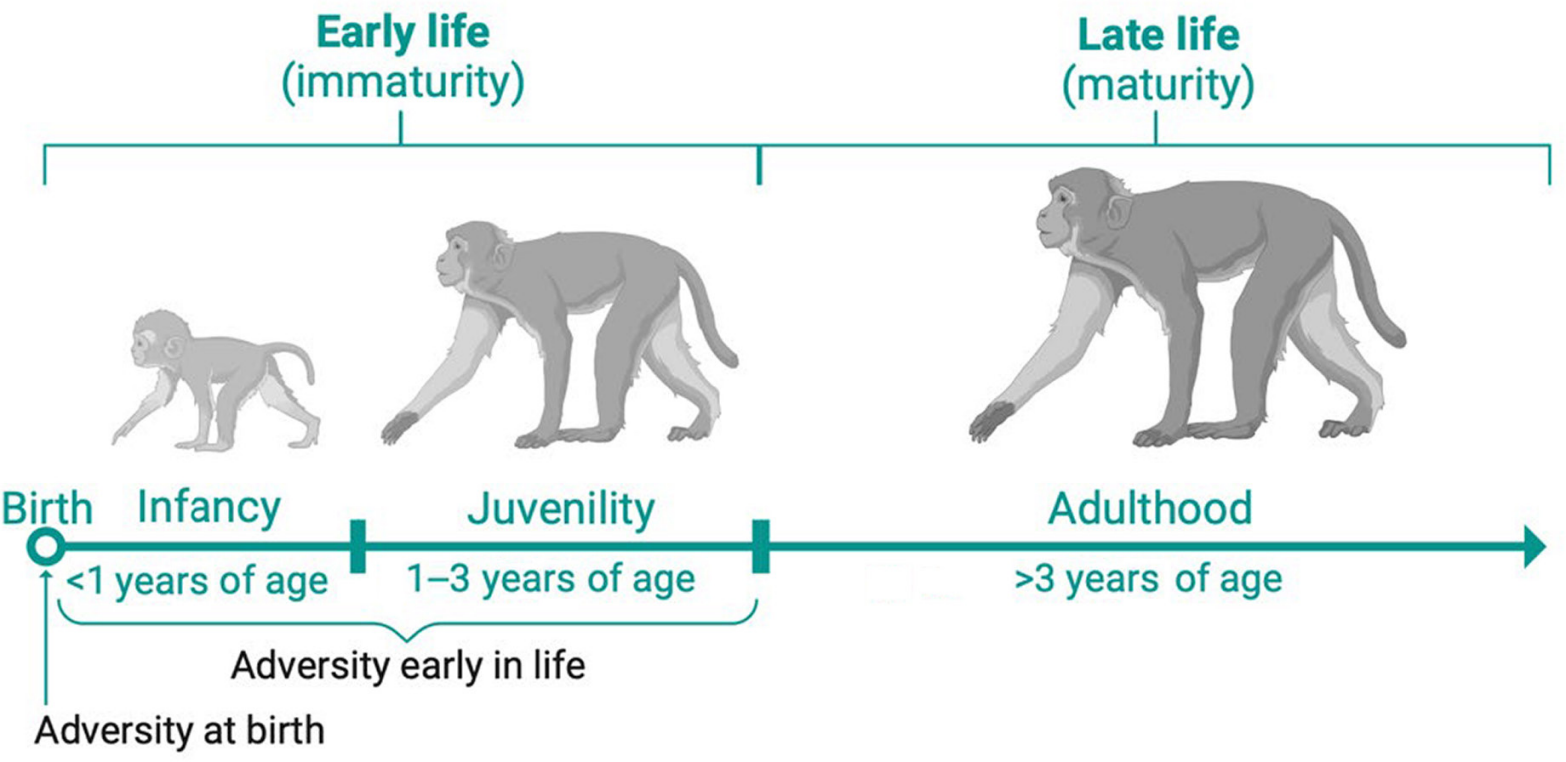
Life stages and early life period of rhesus macaques at Cayo Santiago. To address short‐term effects of adversities on survival, we evaluated associations between sources of adversity at birth and infant survival. To address long‐term effects of adversities on survival, we evaluated associations between sources of adversity early life and adult survival.

To address the short‐term effects of adversities on survival, we first evaluated four sources of adversity at birth: impending maternal death, maternal primiparity, a major hurricane environment, and population density (Table 1). We focused this analysis on infants (<1 year of age) as infancy is the most vulnerable life stage during immaturity in this population (Blomquist, 2013; Hernández‐Pacheco et al., 2013; Hernández‐Pacheco, Delgado, et al., 2016; Hoffman et al., 2010; Figure 2). In Cayo Santiago, female reproduction is energetically costly and poor maternal body condition is associated with infant mortality potentially due to poor lactation performance (Hoffman & Maestripieri, 2012). Because data on body condition are not available, we used impending maternal death as a proxy for poor maternal body condition. Thus, we assumed that a mother who died within 12 months of her offspring's birth was not experiencing optimal body condition at delivery. Cayo Santiago has no predators, and thus, the cause of death for reproductive females is thought to be mostly illness and disease. Maternal effects may also manifest through age‐effects, but a recent study in Cayo Santiago found that maternal age may act indirectly through its influence on offspring body mass (Lee et al., 2020). It is also known that rhesus macaque daughters from primiparous mothers show a lower body mass likely due to reduced milk volume (Pittet & Hinde, 2023). Thus, we incorporated primiparity as another adversity at birth. In addition, Cayo Santiago is located in the “Hurricane Alley” of the Atlantic Ocean and evidence of hurricane‐induced detrimental effects on the population is starting to accumulate (reduced fertility: Morcillo et al., 2020; increased adult mortality: Testard et al., 2021; immunological aging: Watowich et al., 2022). During our study period, Cayo Santiago was affected by three major hurricanes (Category ≥3): Hugo (Category 3, windspeed: 201 km/h) on September 18, 1989; Georges (Category 3, windspeed: 185 km/h) on September 21, 1998; and Maria (Category 4, windspeed: 220 km/h) on September 20, 2017. Each hurricane event reduced tree canopy by 60%–90% (Morcillo et al., 2020; Testard et al., 2021). Although ad libitum food was accessible 1–3 days after each hurricane event, the population is known to spend 50% of daily feeding time on natural vegetation (Marriott et al., 1989). Finally, we considered increased population density as another potential ecological adversity. Competition for access to food, even under food provisioning, has been described in Cayo Santiago in part because food corrals represent clumped resources (Balasubramaniam et al., 2014; Bercovitch & Berard, 1993). Thus, there is both inter‐group competition and intra‐group competition for access to food. As solid food starts being consumed during infancy, infants can experience such competition directly; however, increased density may also impact their mothers through food competition and increased aggression (Balasubramaniam et al., 2014; Maestripieri & Georgiev, 2016).

**TABLE 1:** Adversity sources and criteria.

Adversity source	Criterion
At birth	
Impending maternal death	The mother of the focal individual died within 1 year of the individual's birth
Maternal primiparity	The mother of the focal individual is primiparous (the individual is a firstborn offspring)
Major hurricane environment	The focal individual was born within 1 year of a hurricane event
Population density	Number of adult females at the onset of the focal individual's birth season
Early in life	
Competing younger sibling	The mother of the focal individual had an offspring in the following birth season consecutive to the individual's season
Maternal loss	The mother of the focal individual died or was permanently removed from the population within 3 years of the individual's birth
Maternal primiparity	The mother of the focal individual is primiparous (the individual is a firstborn offspring)
Major hurricane	The focal individual experienced a major hurricane event in their first 3 years of life
Population density	Number of adult females at the onset of the focal individual's birth season

With this information, we assigned all infants three binary variables describing whether they experienced impending maternal death, maternal primiparity, and a hurricane environment, as well as the numerical variable for density, at birth (Table 1). Those individuals whose mother died within their first year of life were classified as experiencing impending maternal death at birth. Monkeys whose mothers were permanently removed from the population within their first year of life were not included in this analysis due to missing information to define impending maternal death for that infant. Primiparity was determined using the mother's reproductive history. Firstborn monkeys were classified as having a primiparous mother. Monkeys being born within a year of a major hurricane event were classified as experiencing a hurricane environment at birth. Thus, we assumed exposure a year after the event had negligible effects on survival. We defined density as the total number of adult females (≥3 years of age) alive at the onset of each focal individual's birth season. We used the annual number of adult females given its influence on population dynamics. In this population, the annual number of adult females is negatively associated with the annual mean fertility of females (Hernández‐Pacheco et al., 2013). We determined density at the onset of each birth season because Cayo Santiago monkeys exhibit reproductive synchrony with 73% of births occurring in a 3‐month period (Hernández‐Pacheco, Rawlins, et al., 2016), and thus, such density represents more accurately the experienced density of mothers post‐conception as opposed to the density at birth due to the potential variation in density caused by culling events (permanent removal), especially late in the birth season (Luevano et al., 2022). Our focal individuals included all infants born in Cayo Santiago between 1973 and 2018, except for those whose mothers were permanently removed (as described above; Table 2).

**TABLE 2:** Frequency of individuals exposed to each adversity source.

	No. of exposed individuals		Frequency	
	Males	Females	Males	Females
Adverse condition at birth (*n*_males_: 4435; *n*_females_: 4195)				
Impending maternal death	192	184	0.04	0.04
Maternal primiparity	745	760	0.17	0.18
Major hurricane environment	213	199	0.05	0.05
Population density (high)	1167	1123	0.26	0.27
Adverse condition early in life (*n*_males_: 2142; *n*_females_: 2229)				
Competing younger sibling	1520	1606	0.71	0.72
Maternal loss	282	286	0.13	0.13
Maternal primiparity	408	424	0.19	0.19
Major hurricane	550	586	0.26	0.26
Population density (high)	563	562	0.26	0.25

To evaluate whether each of these adversities at birth had a short‐term effect on survival, we tracked each newborn over time and recorded the age at death or age at right censorship (i.e., 1 year of age, removal). Monkeys that survived to age one were treated as censored individuals who at least survived the infancy period. For each sex, we used the Kaplan–Meier estimator and the log‐rank test to estimate and compare survival functions followed by the Cox proportional hazards regression to estimate and compare hazard ratios. As the Kaplan–Meier estimator can only consider groups as explanatory variables, we binarized density. For this, we evaluated the observed distribution of the annual number of adult females at the onset of each birth season. Individuals born in a year where the number of adult females was above the 3rd quartile of the distribution (≥349 adult females) were classified as experiencing high density. This is in line with prior reports on density effects on births (Hernández‐Pacheco & Steiner, 2017). To test whether experiencing any of the four sources of adversity at birth increased mortality risk during infancy, we fitted mixed‐effects Cox proportional hazards models using each adversity as a fixed effect. In this analysis, adult female density was kept as a numerical variable. To account for unobserved maternal effects, we included maternal ID as a random intercept. We tested correlations among adversities using phi‐coefficients, point‐biserial correlations, and Spearman correlations (Tables S1 and S2). Given no strong correlations (−.5 < *r* < .5), we added all variables to a global model. As maternal age and infant mortality may be associated (Blomquist, 2013), we also considered another set of Cox regression models that controlled for maternal age using a linear and a quadratic effect of age but did not include primiparity due to a strong correlation (Appendix S3). Because the proportional hazards (PH) assumption, i.e., the relation between the adversity effect and the time to death was not constant over time, was not met for the density covariate in our initial global models for male and female infants, we extended our Cox analyses using time‐varying covariates by stratifying density into two age periods (males: birth to 2.3 months and 2.3–12 months; females: birth to 0.84 months and 0.84‐12 months) following visual inspection of the estimated coefficient across time (Therneau et al., 2021; Zhang, Reinikainen, et al., 2018; Appendices S4 and S5). Such approach allows us to describe, for instance, how the association between density at birth and infant survival changes across different age periods during infancy (Lee et al., 2020). Similarly, the PH assumption was not met for the primiparity covariate in the global model for female infants, and thus, we stratified primiparity into two age periods (birth to 9.2 months and 9.2–12 months) following visual inspection of the estimated coefficient across time (Appendix S5). These new time‐varying models met all assumptions for proportional hazards. We ran all models in R version 4.1.2 (R Core Team, 2021) using the packages *survival* (Therneau, 2021) and *coxme* (Therneau, 2022).

### 2.3 Cumulative adversity at birth and infant survival

To evaluate whether the accumulation of adversities at birth was associated with infant survival, we constructed a cumulative adversity index defined as the total number of adversities an individual experienced at birth (Morrison et al., 2023; Tung et al., 2016; Zipple et al., 2019). For this analysis, we used the binarized definition for “high density” previously described. We used sex‐specific Kaplan‐Meier estimators and the log‐rank tests, and fitted mixed‐effects Cox proportional hazards models using the cumulative adversity index as a fixed effect and included maternal ID as a random intercept. Monkeys who survived to age one were treated as censored individuals who at least survived the infancy period. Given that the PH assumption was not met for the cumulative adversity covariate in both male and female infant global models, we extended our analyses using time‐varying covariates by stratifying cumulative adversity into two age periods (birth to 10.8 months and 10.8‐12 months) following visual inspection of the estimated coefficient across time (Therneau et al., 2021; Zhang, Reinikainen, et al., 2018; Appendices S4 and S5). These new time‐varying models met all assumptions for proportional hazards.

### 2.4 Adversity early in life and adult survival

We extended our previous analysis to five sources of adversity early in life: competing younger sibling, maternal loss, maternal primiparity, major hurricanes, and population density. We defined early life as the period from birth to the end of immaturity (<3 years of age; Luevano et al., 2022; Table 1; Figure 2). In this population, many reproductive females produce offspring in consecutive annual birth seasons because they may resume their menstrual cyclicity and mate after 6 months of birth (Hernández‐Pacheco, Delgado, et al., 2016; Maestripieri & Hoffman, 2012). As a result, many focal individuals experience a younger sibling, and this may produce stress given reduced maternal care and increased aggression (Devinney et al., 2001, 2003; Table 2). A competing younger sibling was identified as a sibling being born during the consecutive birth season after the focal individual's own birth season (i.e., 1 year apart, approximately). Maternal primiparity and population density were defined as described above. Maternal loss, however, was defined through maternal death or permanent removal during immaturity. Similarly, experiencing a hurricane was determined by whether the individual experienced a major hurricane event during immaturity. With this information, we assigned all individuals four binary variables describing whether they experienced a competing younger sibling, maternal loss, primiparity, and hurricanes, and the numerical variable of density. Our focal individuals included all rhesus macaques born in Cayo Santiago between 1973 and 2018 that survived to adulthood (Table 2). We monitored all individuals until death, removal, or the end of the study in 2022.

To evaluate whether early life adversity was associated with adult survival, we tracked each adult individual (≥3 years of age) over time and recorded the age at death or age at right censorship (i.e., removal or end of study). For each sex, we used the Kaplan–Meier estimator and the log‐rank test to estimate and compare survival functions followed by a mixed‐effects Cox proportional hazards regression to estimate and compare hazard ratios. As described above, we estimated survival functions for adult monkeys experiencing each adversity relative to those who did not experience such adversity. To test whether experiencing any of the five sources of adversity early in life increased mortality risk during adulthood, we fitted sex‐specific mixed‐effects Cox proportional hazards models using each adversity as a fixed effect and maternal ID as a random intercept. Because the PH assumption was not met for the hurricane covariate in our initial global model for male adults, we extended the Cox analysis using time‐varying covariates by stratifying the hurricane covariate into two age periods (3–8 and >8 years) following visual inspection of the estimated coefficient across time (Therneau et al., 2021; Zhang, Reinikainen, et al., 2018; Appendix S4). Similarly, the PH assumption was not met for the maternal loss covariate in the global model for female adults, and thus, we stratified maternal loss into two age periods (3–18 and >18 years) following visual inspection of the estimated coefficient across time (Appendix S5). These new time‐varying models met all assumptions for proportional hazards.

### 2.5 Cumulative adversity early in life and adult survival

To evaluate whether the accumulation of adversities early in life was associated with adult survival, we constructed a cumulative adversity index as described above. For this analysis, we tracked each adult individual over time and recorded the age at death or age at right censorship. We used sex‐specific Kaplan‐Meier estimators and the log‐rank tests, and fitted mixed‐effects Cox proportional hazards models using the index as a fixed effect and included maternal ID as a random intercept. Given that the PH assumption was not met for the cumulative adversity covariate in the male adult initial global model, we extended this analysis to time‐varying covariates by stratifying cumulative adversity into two age periods (3–10 and >10 years) following visual inspection of the estimated coefficient across time (Therneau et al., 2021; Zhang, Reinikainen, et al., 2018; Appendix S4). This new time‐varying model met all assumptions for proportional hazards.

## 3 RESULTS

### 3.1 Short‐term effects of adversity

**FIGURE 3:**
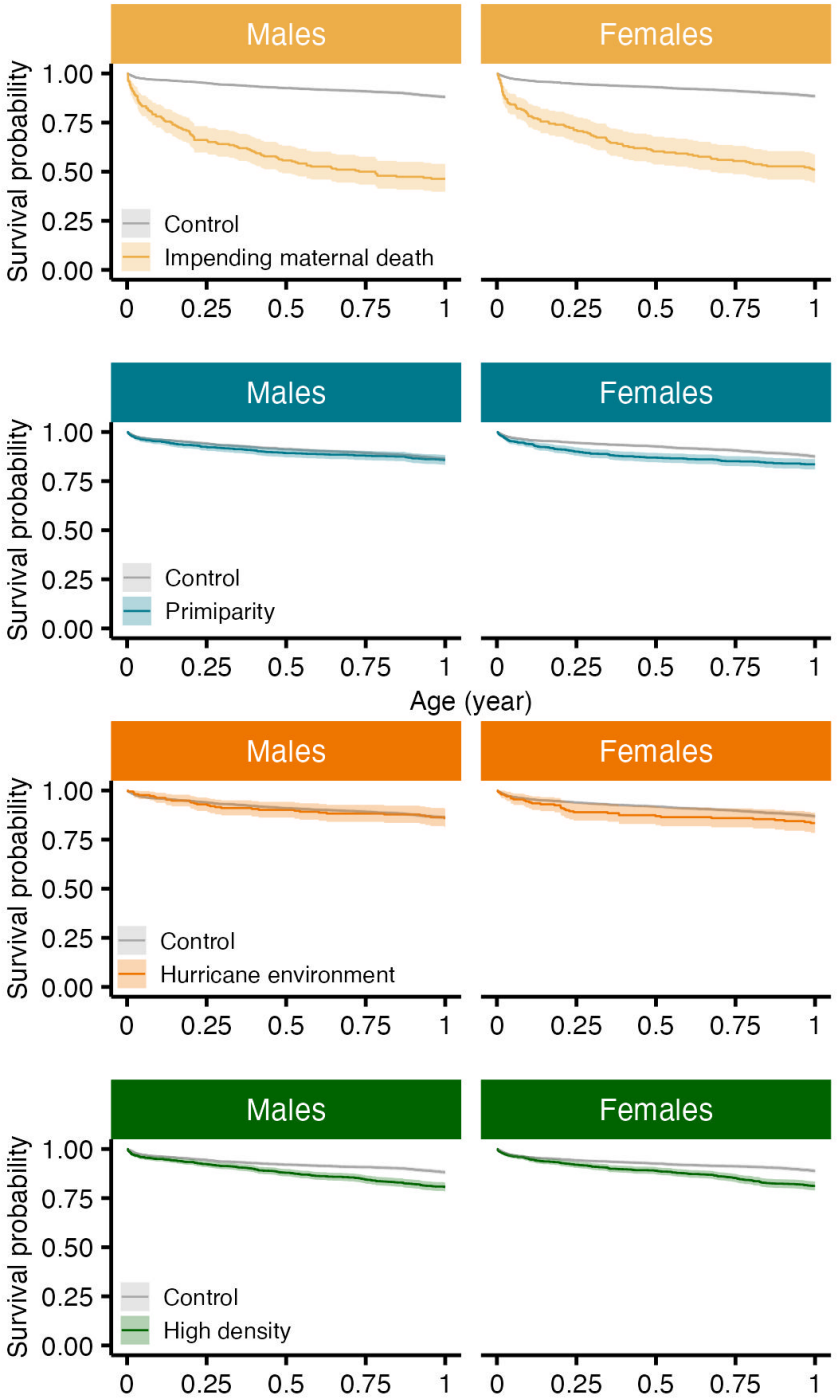
Survival curves for male (left) and female (right) infant rhesus macaques experiencing different sources of adversity at birth. Ribbons represent 95% confidence intervals.

**FIGURE 4:**
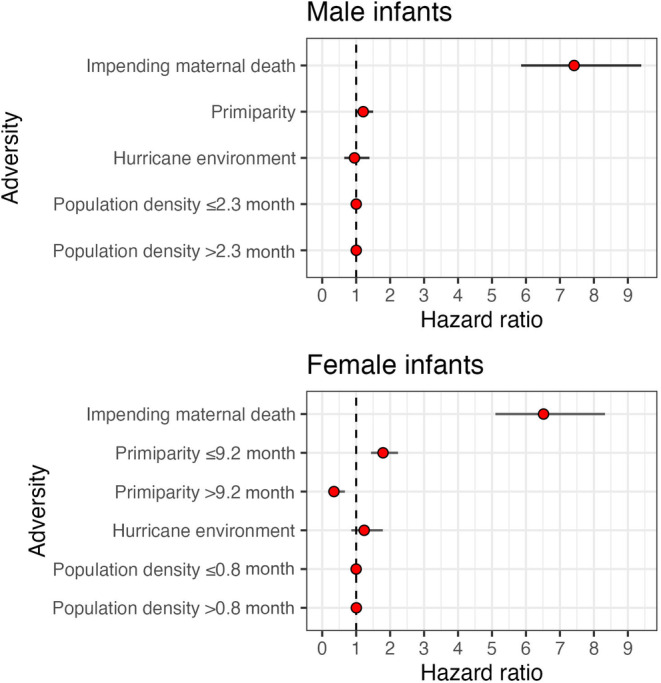
Hazard ratios for male (top) and female (bottom) infant rhesus macaques experiencing different sources of adversity at birth. Bars represent 95% confidence intervals. The dashed line represents a HR = 1.0 (95% CI overlapping 1 indicate no significance).

The survival of male infants was significantly reduced for those who experienced impending maternal death and increased density at birth (*p* < .01; Figure 3). Males experiencing impending maternal death who also died in the population had a median age at death of approximately 0.25 years (95% CI: 0.00, 9.63), or 3 months, in contrast to males who did not experience impending maternal death with a median age at death of 2.09 years (0.01, 21.37). Male infants experiencing impending maternal death were more than 7 times as likely to die relative to males that did not experience the adversity (HR = 7.42, 95% CI: 5.86, 9.39; *n* = 4435; Table 3; Figure 4). Male infant survival was less likely for those born in increased density seasons, but this relationship was significant only after 2.3 months of life (Table 3). Increasing population density in increments of one additional adult female increased male infant mortality risk by 0.2% after reaching 0.19 years, or 2.3 months of age (HR = 1.002; 95% CI: 1.00, 1.004; Table 3; Figure 4). We found no associations between the survival of male infants and maternal primiparity or hurricane environment (Table 3). After controlling for maternal age, these associations hold (Appendix S3). Young and old maternal ages increased mortality risk (Appendix S3).

**TABLE 3:** Hazard ratio estimated from Cox regression models testing associations between adversity at birth and rhesus macaque infant survival.

	Hazard ratio	SE	95% CI
Males (*n* = 4435)			
Individual effects			
Impending maternal death	**7.417**	**0.121**	**5.857, 9.394**
Maternal primiparity	1.207	0.111	0.970, 1.502
Major hurricane environment	0.951	0.195	0.649, 1.392
Population density ≤2.3 months	1.001	0.001	1.000, 1.003
Population density >2.3 months	**1.002**	**0.001**	**1.000**, **1.004**
Cumulative effects			
Cumulative Adversity Index ≤10.8 months	**1.892**	**0.063**	**1.674**, **2.138**
Cumulative Adversity Index >10.8 months	**0.538**	**0.213**	**0.354**, **0.816**
Females (*n* = 4195)			
Individual effects			
Impending maternal death	**6.517**	**0.125**	**5.101**, **8.327**
Maternal primiparity ≤9.2 months	**1.791**	**0.112**	**1.437**, **2.232**
Maternal primiparity >9.2 months	**0.346**	**0.337**	**0.179**, **0.669**
Major hurricane environment	1.237	0.187	0.857, 1.784
Population density ≤0.8 month	0.999	0.001	0.997, 1.001
Population density >0.8 month	**1.005**	**0.001**	**1.003**, **1.008**
Cumulative effects			
Cumulative Adversity Index ≤10.8 months	**2.189**	**0.065**	**1.926**, **2.486**
Cumulative Adversity Index >10.8 months	**0.303**	**0.258**	**0.183**, **0.503**

*Note*: Bold 95% CIs indicate significance at .05 level (i.e., do not overlap with 1).

**FIGURE 5:**
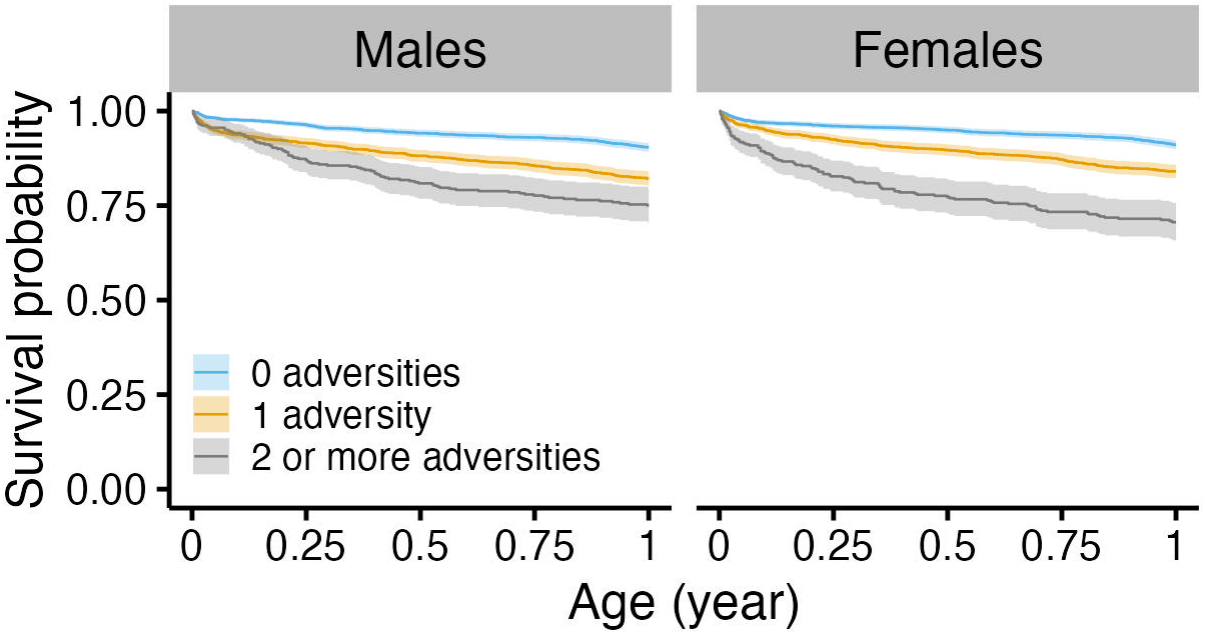
Survival curves for male (left) and female (right) infant rhesus macaques experiencing cumulative adversity at birth. Ribbons represent 95% confidence intervals.

The survival of male infants was also associated with the accumulation of adversities at birth (*p* < .001, Figure 5). Males experiencing 1 adversity at birth who also died in the population showed a median age at death of 1.13 years (0.01, 19.03), while those experiencing 2 or more adversities showed a median age at death of 0.56 years (0.01, 17.93). This is in contrast to males who did not experience any adversity at birth with a median age at death of 4.45 years (0.02, 22.03). However, the association between cumulative adversity and male infant survival was not constant over time. Experiencing an additional adversity at birth increased male infant mortality risk by 89.2% until approximately 0.90 years, or 10.8 month of life (HR = 1.89; 95% CI: 1.67, 2.14; *n* = 4435; Table 3). After 10.8 months, male infants experiencing an additional adversity showed a 46.2% reduction in mortality risk (HR = 0.54; 95% CI: 0.35, 0.82; Table 3).

The survival of female infants was associated with impending maternal death, maternal primiparity, and increased density at birth (*p* < .01; Figure 3). Females experiencing impeding maternal death who also died in the population showed a median age at death of 0.35 years (95% CI: 0.01, 15.69), or approximately 4.2 months, in contrast to females who did not experience impeding maternal death with a median age at death of 2.08 years (0.01, 22.76). Female infants experiencing impending maternal death were more than 6 times as likely to die relative to females that did not experience the adversity (HR = 6.52; 95% CI: 5.10, 8.33, *n* = 4195; Table 3). Female infant survival was less likely for those with maternal primiparity, but this relationship sustained until 0.77 years, or 9.2 months of age. Females having a primiparous mother who also died in the population showed a median age at death of 1.35 years (0.01, 22.79), in contrast to females who did not experience a primiparous mother with a median age at death of 1.98 years (0.01, 22.69). Female infants experiencing maternal primiparity showed a 79.1% increase in the risk of dying until reaching approximately 0.77 years, or 9.2 months of age (HR = 1.79, 95% CI: 1.44, 2.23; Table 3). After 9.2 months of age, having a primiparous mother significantly reduced mortality risk by 65.4% (HR = 0.35, 95% CI: 0.18, 0.67; Table 3). Similarly, female infant survival was less likely for those born into an increased density season, but this relationship was significant after 0.07 years, or 0.84 months, of age. Increasing population density in increments of one additional adult female increased female infant mortality risk by 0.50% after reaching approximately 0.07 years, or 0.84 months, of age (HR = 1.005, 95% CI: 1.00, 1.01; Table 3). We found no evidence of associations between the survival of female infants and major hurricane environments. After controlling for maternal age, these relationships hold (Appendix S3). Young and old maternal ages increased mortality risk (Appendix S3).

The survival of female infants was also associated with the accumulation of adversities (*p* < .001, Figure 5). Female infants experiencing 1 adversity who also died in the population showed a median age at death of 1.17 years (0.01, 21.00), while those experiencing 2 or more adversities showed a median age at death of 0.59 years (0.01, 21.71), or approximately 7.1 months. This is in contrast to females who did not experience any adversity at birth with a median age at death of 4.03 years (0.02, 23.46). However, the association between cumulative adversity and female infant survival was not constant over time. Females experiencing an additional adversity were twice as likely to die until approximately 0.90 years, or 10.8 months, of age relative to female infants who did not experience cumulative adversity (HR = 2.19; 95% CI: 1.93, 2.49; *n* = 4195; Table 3). After 10.8 months of age, experiencing an additional adversity significantly reduced female infant mortality risk by 69.7% (HR = 0.30; 95% CI: 0.18, 0.50; Table 3).

### 3.2 Long‐term effects of early life adversity

**FIGURE 6:**
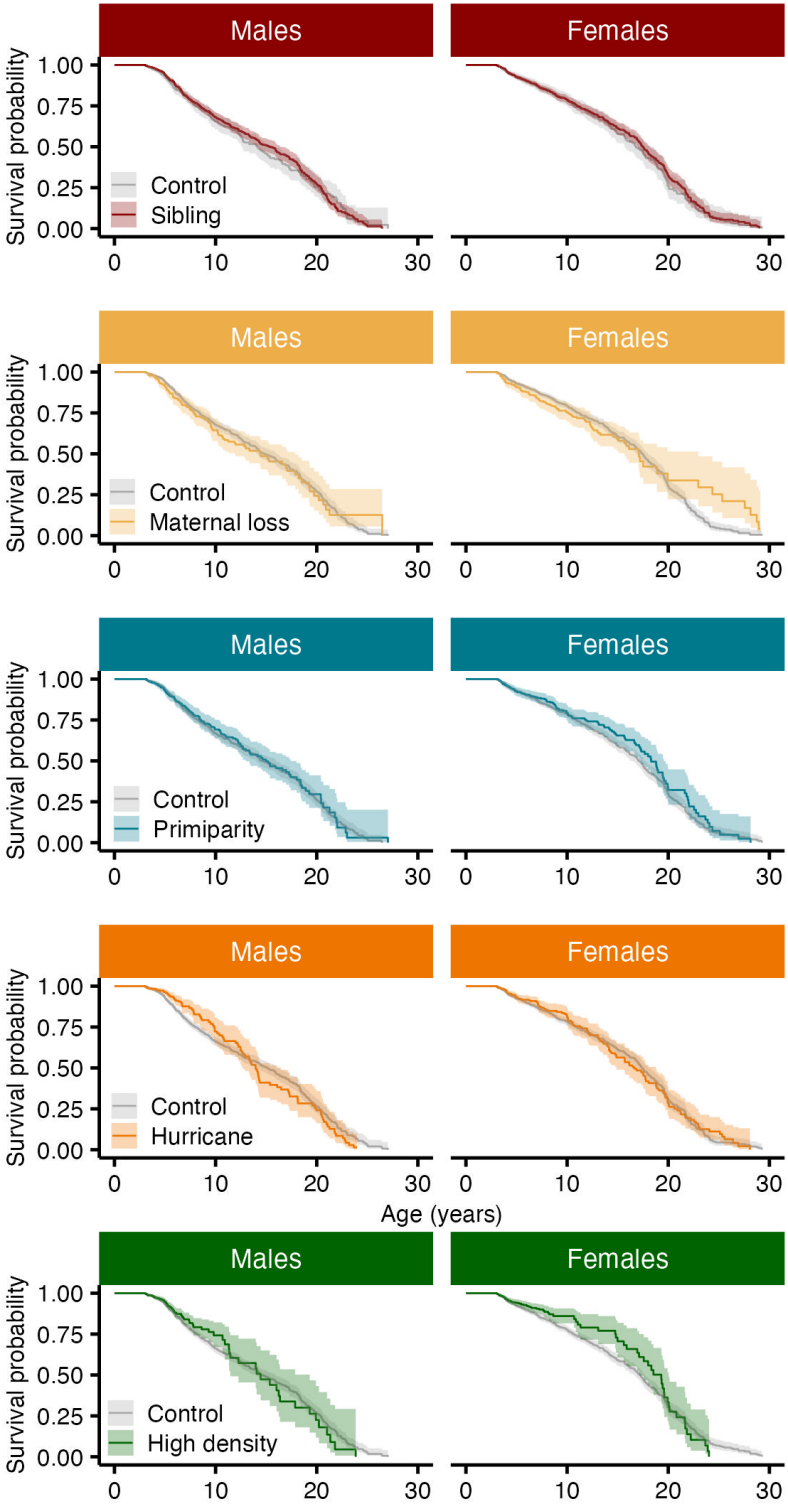
Survival curves for male (left) and female (right) adult rhesus macaques experiencing different sources of adversity early in life. Ribbons represent 95% confidence intervals.

**FIGURE 7:**
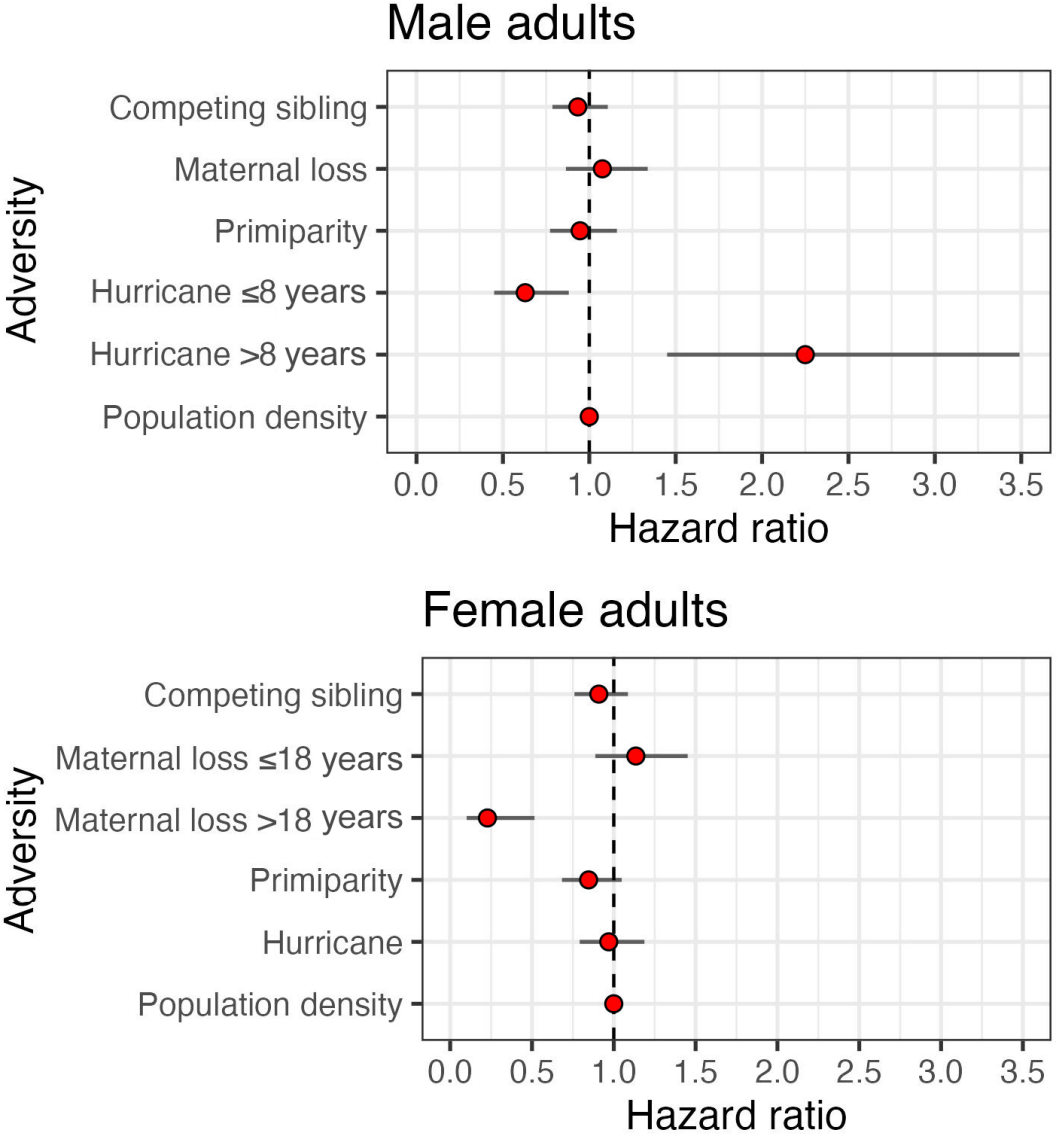
Hazard ratios for male (top) and female (bottom) adult rhesus macaques experiencing different sources of early life adversity. Bars represent 95% confidence intervals. The dashed line represents a HR = 1.0 (95 CI overlapping 1 indicate no significance).

**FIGURE 8:**
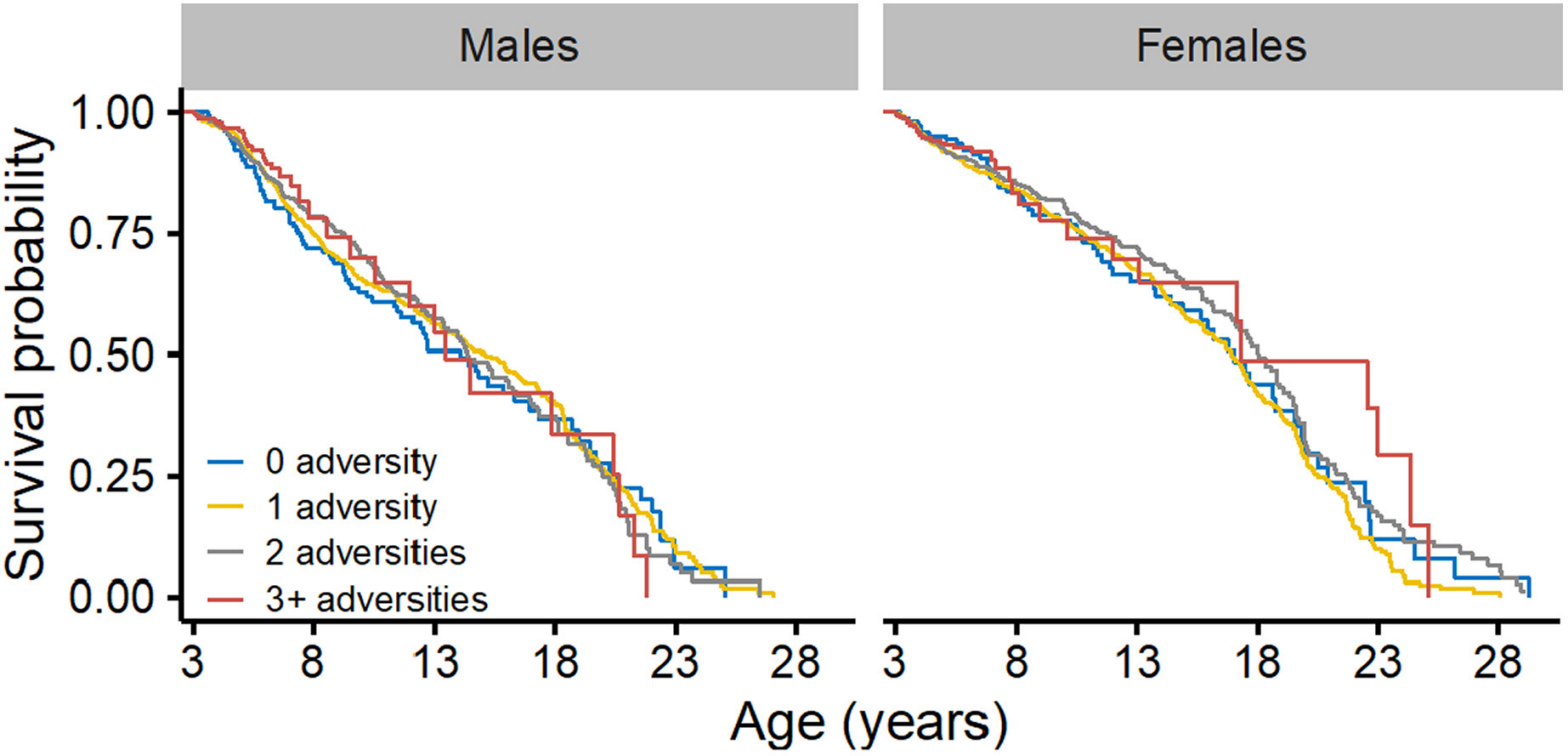
Survival curves for male (left) and female (right) adult rhesus macaques experiencing cumulative adversity early in life. For visual comparison, 95% confidence intervals are not shown.

The survival curve functions of male adults experiencing early life adversity were not significantly different to those from males who did not experience adversity early in life (*p* > .05, Figure 6). However, the mortality hazard was associated with major hurricanes. Male adults experiencing a major hurricane event early in life who also died in the population had a median age at death of 10.51 years (95% CI: 3.10, 22.82), in contrast to males who did not experience a major hurricane event with a median age at death of 7.88 years (3.37, 22.88). Yet, the association between hurricanes and male adult survival was not constant over time. Male adults who experienced a major hurricane early in life showed a 37.1% reduction in mortality risk up to 8 years of age (HR = 0.63; 95% CI: 0.45, 0.88; *n* = 2142; Table 2; Figure 7), relative to males that did not experience a major hurricane event. After 8 years of age, however, these male adults were twice as likely to die compared to males that did not experience this adversity (HR = 2.25, 95% CI: 1.45, 3.49; Table 2; Figure 7). We found no associations between male adult survival and the presence of a competing younger sibling, maternal loss and primiparity, and density (Table 4). Similarly, the survival curve functions of male adults experiencing early life cumulative adversity were not significantly different to that of males who did not experience adversities (*p* > .05, Figure 8). However, the hazard ratio of male adults was associated with cumulative adversities early in life, but such association was not constant over time. Experiencing one additional adversity early in life reduced mortality risk by 13.4% up to 10 years of age (HR = 0.87; 95% CI: 0.76, 0.98; *n* = 2142; Table 4) relative to males who did not experience early life adversity. After 10 years of age, however, experiencing one additional adversity early in life significantly increased male adult mortality risk by 37.9% (HR = 1.38; 95% CI: 1.11, 1.71; Table 4).

**TABLE 4:** Hazard ratio estimated from Cox regression models testing associations between early life adversity and rhesus macaque adult survival.

	Hazard ratio	SE	95% CI
*Males* (*n* = 2142)			
Individual effects			
Competing sibling	0.933	0.087	0.787, 1.107
Maternal loss	1.076	0.111	0.865, 1.338
Maternal primiparity	0.947	0.104	0.773, 1.160
Major hurricane 3–8 years	**0.629**	**0.172**	**0.449**, **0.881**
Major hurricane >8 years	**2.250**	**0.224**	**1.450**, **3.490**
Population density	1.000	0.000	0.999, 1.001
Cumulative effects			
Cumulative Adversity Index 3–10 years	**0.866**	**0.064**	**0.764**, **0.983**
Cumulative Adversity Index >10 years	**1.379**	**0.111**	**1.109, 1.714**
Females (*n* = 2229)			
Individual effects			
Competing sibling	0.908	0.091	0.759, 1.086
Maternal loss 3–18 years	1.135	0.126	0.887, 1.452
Maternal loss >18 years	**0.228**	**0.416**	**0.101**, **0.515**
Maternal primiparity	0.846	0.109	0.683, 1.048
Major hurricane	0.969	0.103	0.791, 1.187
Population density	1.000	0.001	0.999, 1.001
Cumulative effects			
Cumulative Adversity Index	**0.887**	**0.056**	**0.794**, **0.991**

*Note*: Bold 95% CIs indicate significance at .05 level (i.e., do not overlap with 1).

Likewise for females, the survival curve functions of adults experiencing early life adversity were not significantly different from that of adults who did not experience an adversity (*p* > .05; Figure 6). However, the hazard ratio was associated with maternal loss (Table 4; Figure 7). Female adult survival was more likely for those experiencing maternal loss, but this relationship became significant after 18 years of age. Female adults who experienced maternal loss early in life showed a 77.2% reduction in mortality risk after 18 years of age (HR = 0.23, 95% CI: 0.10, 0.52; *n* = 2229; Table 4; Figure 7). We found no associations between female adult survival and the presence of a competing younger sibling, maternal primiparity, major hurricanes, and density (Table 4; Figure 7). The survival curve function of female adults experiencing early life cumulative adversity was not significantly different to that of females who did not experience adversities (*p* > .05; Figure 8). However, the mortality risk of female adults was associated with cumulative adversities early in life. Experiencing one additional adversity during early life reduced female adult mortality risk by 11.3% at every age (HR = 0.89; 95% CI: 0.79, 0.99; *n* = 2229; Table 4).

## 4 DISCUSSION

Our analysis shows that early life adversity effects on survival are context specific. Rhesus macaques exposed to adversity at birth suffered a significant increase in risk of death during infancy. However, when we considered adult survival, males were negatively affected by both individual and cumulative adversity early in life, in contrast to females. Our study shows how insults early in life can have profound immediate effects on the survival of female cohorts and suggests that affected females who managed to survive into adulthood are robust. Our study also reveals a delayed response of adult males who experienced early life adversity in which negative consequences of adversity appear only at older adult ages.

### 4.1 Short‐term effects of adversities on survival

The survival of infants was significantly associated with impending maternal death. This is expected for altricial species such as rhesus macaques where maternal effects on infant survival are strong (Blomquist, 2013; Hoffman et al., 2010). During early life, mothers serve as the main source of nutrition, as well as the strongest social bond for offspring (Maestripieri & Hoffman, 2012). In particular, we found a higher difference in risk of death among male infants experiencing impending maternal death, relative to males who did not experience it. In our population, maternal investment in male offspring is thought to be greater than in females due to the faster growth rate of male offspring and the positive relationship between the weight of male offspring and their future reproductive output (Bercovitch et al., 2000). Thus, the inability to fulfill the energetic demands of fast‐growing male offspring likely due to poor maternal condition could have led to a higher risk of death among affected males. Although knowledge on maternal death is highly accurate in our population, we acknowledge that the criteria used to define impending maternal death may be combining the effects of poor maternal health and complete absence of the mother in some of our focal infants. However, accurately disentangling these two effects was not possible in our study. Our analysis also shows that female infants born to a primiparous mother experienced an increased risk of death, relative to females who were born to an experienced mother. A recent study on captive rhesus macaques showed that firstborn daughters grew slower during immature ages, and these affected daughters later synthesized less available milk energy (milk energetic density by milk yield) compared to daughters of multiparous mothers (Pittet & Hinde, 2023). Although we did not measure intergenerational effects of primiparity, such related physiological and growth constraints may explain why having a primiparous mother negatively affected female infants. We did not observe a significant relationship between primiparity and infant male survival. Sex‐biased investment during lactation varies with maternal life history in rhesus macaques (Bercovitch et al., 2000). Prior evidence suggests that milk quality in primiparous mothers is biased toward male offspring due to potential mechanisms of sex‐specific regulation of anabolic and growth hormones or metabolic efficiency (Hinde, 2009). Producing a higher‐quality milk for male offspring suggests a flexible reproductive strategy of mothers to cope with high energetic demands of fast‐growing offspring with higher reproductive output (Bercovitch et al., 2000; Hinde, 2007, 2009; Landete‐Castillejos et al., 2005; Robert & Braun, 2012; Trivers & Willard, 1973).

In contrast to maternal sources of adversity, infants being born in a hurricane environment exhibited no difference in risk of death, relative to those born in ordinary environments. This reflects prior survival analyses addressing the effects of ecological sources of early life adversity on lifespan in this population (Luevano et al., 2022). Hurricane‐induced changes in the annual demography of the Cayo Santiago rhesus macaques are mostly driven by suppressed mean female fertility (Morcillo et al., 2020). It could be the case that mothers who are able to successfully produce viable offspring during extreme climate environments possess a higher quality that can be passed on to their offspring (our subjects), buffering against mortality risk (Jenouvrier et al., 2015). However, infants who experienced increased density of adult females at birth showed a higher mortality risk. Although the risk of death was similar for males and females, the negative effects of increased density were observed earlier in the life of affected female infants. In Cayo Santiago, high density of adult females can lead to increased inter‐ and intra‐group competition for access to food (Bercovitch & Berard, 1993; Sterck et al., 1997). In other rhesus macaque populations, high density has been associated to increased levels of cortisol, a hormone often used to measure stress levels (Dettmer et al., 2014), and increased aggressive interactions between female kin and non‐kin (Judge & De Waal, 1997). Variation in sex‐specific infant susceptibility to high population density can be further explained by antagonistic encounters in environments with limited resources. In several macaque species, female infants are often more susceptible to mortality from adult female attacks than male infants (dispersing sex) as a potential mechanism to reduce future competition within the social group (toque macaques: Dittus, 1979; bonnet macaques: Silk et al., 1981). In our study population, adult females tended to threaten female infants more than male infants (Berman, 1980). Thus, increased aggression among females and their daughters during high female density years likely contributed to the observed negative density effect earlier in the life of females, in contrast to males.

The accumulation of adversity at birth also had negative effects on infant mortality risk, but the risk resulted higher among affected female infants. Yet, more than 50% of female infants were alive at 1 year of age regardless of the number of adversities they experienced (Figure 5). This is contrary to infants experiencing impending maternal death and thus suggests that mortality risk during infancy is mainly driven by the type of adversity and not their accumulation at birth for both sexes. For these rhesus macaques, maternal investment remains the main driver of infant survival.

### 4.2 Long‐term effects of early life adversities on survival

Our analysis demonstrates that individual insults at birth strongly predict the survival of infants, but contrary to our predictions, many of these effects do not translate into adulthood. In addition, we found that the male adult response to early life insults differed from that of females; thus, sex‐specific selection pressures on life histories may be acting on this population. When addressing individual adversity effects, the survival of male adults was only associated with major hurricanes. Male adults experiencing a major hurricane early in life showed an initial reduction in mortality risk. However, risk increased at older ages. This response to extreme climates early in life was unexpected given the absence of an association with infant mortality. It is possible that experiencing a hurricane event first‐hand, as opposed to being born into the aftermath of it, may pose different stressful environments. However, recent work on the role of hurricanes on this rhesus macaque population suggests such extreme events play an important role on variability in individual life courses (Diaz et al., 2023). Here, we provide important evidence of the long‐lasting effects that extreme climatic events early in life have on rhesus macaque males. In contrast, female adult survival was associated with maternal loss, but such relation was positive. Affected female adults showed a significant reduction in mortality risk at very old ages, relative to unaffected ones. Our findings support prior evidence of the high resiliency of female adults in this population, including resilience to extreme climatic events (Morcillo et al., 2020). On the other hand, male adults with a higher accumulation of adversities early in life showed an initial reduction in mortality risk, followed by a significant increase in risk at older ages. In contrast, female adults with a higher accumulation of adversities showed a reduced mortality risk at any given age. Thus, our analysis on adult survival supports hypotheses on detrimental effects of cumulative adversity on male adult survival, but this does not apply to females who showed a greater longevity when experiencing an additional adversity early in life.

### 4.3 Implications to life history theory

Our study contributes to recent reports arguing that the link between early life adversity and increased risk later in the life of primates may not be universal (Morrison et al., 2023), and stimulates several questions regarding the role of early life adversities as drivers of the evolution of primate life histories. Cayo Santiago rhesus macaques live in a naturalistic environment, yet many ecological variables are controlled. In this regard, this population can be used to test mechanistic questions that can shed light into wild populations. In particular, our findings raise questions regarding the contribution of viability selection (Douhard et al., 2014) and life history trade‐offs (Stearns, 1989) as mechanisms shaping the life history of primate populations subjected to harsh conditions early in life.

Viability selection and individual heterogeneity likely play a major role in our population. Affected individuals surviving into adulthood may possess higher‐quality traits compared to those dying at immature ages (Douhard et al., 2014). Female infants showed vulnerability to multiple adversities at birth; however, females that survived to adulthood but experienced adversities or accumulated them during the early life period (immaturity; Figure 2) showed a reduced risk. In contrast, male infants showed vulnerability to a lower number of adversities at birth, but those who survived to adulthood were negatively affected by both early life individual and cumulative adversity. Although our analysis does not directly compares males to females, our findings suggest that females may be experiencing greater viability selection than males, while maternal effects may be buffering male infant mortality in this primate population. Such observation contrast with prior studies suggesting that male mammals are under stronger viability selection, and thus, only males are likely to retain the phenotypes with best fit at old ages (Gamelon et al., 2014; Morrison et al., 2023). Our study mirrors conclusions from a recent study in wild gorillas in which authors demonstrated that the link between early life adversity and increased risk later in life is not widespread and that some primates may be resilient to mortality consequences of early life adversity (Morrison et al., 2023). Our study contributes to this literature by showing that similar processes also occur in the rhesus macaques of Cayo Santiago.

Life history trade‐offs can also play a critical role on adult survival in our primate population. Evidence of early life adversity effects on Cayo Santiago rhesus macaque female reproduction suggested that adversity‐affected females ensure their future reproductive potential by allocating more energy to growth or maintenance processes at younger adult ages at the expense of reproduction (Luevano et al., 2022). This strategy among affected females may be driving the lack of negative associations between adversity early in life and female adult survival. Such lack of associations may result from adaptive physiological mechanisms acting during development that mainly favor survival‐enhancing traits at the cost of reproduction‐enhancing traits (Cooper & Kruuk, 2018; Metcalfe & Monaghan, 2001). Although still unexplored, our findings may reflect sex‐specific life strategies of a promiscuous, sexually dimorphic, primate species: females may allocate more energy toward survival to ensure future reproduction, while males may invest more energy toward reproduction at the expense of long‐term survival in response to early life adversity.

### 4.4 Future directions

We provide further insight into factors that shape primate lifespans by demonstrating differential effects of early life adversity on male and female rhesus macaque survival at different life stages, but the underlying mechanisms shaping these patterns remain unexplored. For example, the social environment is critical for primates; thus, future work must consider social factors as both early life adversity or enhancement and late life mediators. It is well known that social status can bring forth forms of resources and competition (Georgiev et al., 2015; Sapolsky, 2005) and can thus influence glucocorticoid levels (Cavigelli & Caruso, 2015; Gesquiere et al., 2011; Rosenbaum et al., 2020; Sapolsky, 2005; Zhang, Cui, et al., 2018). On the other hand, cohesive social groups may provide social support that buffers against the effect of maternal loss (Morrison et al., 2021), and if social capital is passed on from mother to offspring, offspring from well‐integrated mothers could present a survival advantage, relative to those from poorly integrated mothers (Silk et al., 2009). Following the most intense hurricane in the history of Cayo Santiago, changes in behavior involving an increase in affiliative social connections were observed as individuals became more tolerant of conspecifics (Testard et al., 2021). Thus, social support may also be a fundamental mechanism that gregarious primates can adopt to cope with years of extreme ecological adversity, potentially masking expected effects later in life. Observing early life adversity effects on a provisioned primate population with no seasonal changes in food availability also raises important questions regarding the role that calorie deficit early in life has as a driver of developmental constrains that result in poor adult health and survival. Finally, other adaptive behaviors (e.g., foraging timing; Mainwaring et al., 2023) must also be addressed to fully understand how affected individuals may mitigate the adversity they experienced early in life that likely result in the different observed responses across primate populations.

## AUTHOR CONTRIBUTIONS

**Stephanie J. Gonzalez:** Conceptualization (equal); data curation (lead); formal analysis (lead); investigation (equal); methodology (equal); validation (equal); visualization (equal); writing – original draft (equal); writing – review and editing (equal). **Anthony J. Sherer:** Data curation (supporting); formal analysis (supporting); investigation (supporting); validation (supporting); writing – review and editing (supporting). **Raisa Hernández‐Pacheco:** Conceptualization (equal); funding acquisition (lead); investigation (equal); methodology (equal); project administration (lead); resources (lead); supervision (lead); validation (equal); visualization (equal); writing – original draft (equal); writing – review and editing (equal).

## CONFLICT OF INTEREST STATEMENT

We declare no conflict of interest.

## Supporting information


Appendix S1.

## Data Availability

Data and R codes are available in Dryad (https://doi.org/10.5061/dryad.41ns1rnmj).
