# Supplementary material for: Differential effects of early life adversity on male and female rhesus macaque lifespan
Source: Ecol Evol. 2023 Nov 5;13(11):e10689. doi: 10.1002/ece3.10689 (PMC10626128; doi:10.1002/ece3.10689)
Supplement: Supplementary file 1 — Appendix S1. [file ECE3-13-e10689-s001.docx]

**Supporting Information**

**Differential effects of early life adversity on male and female rhesus macaque lifespan**

Stephanie J Gonzalez^1,2^, Anthony J Sherer^1^, and Raisa Hernández-Pacheco^1^

^1^California State University-Long Beach, Department of Biological Sciences, 1250 N Bellflower Blvd Long Beach, CA 90840-0004; ^2^Department of Ecosystem Science and Management, Pennsylvania State University, University Park, PA 16802

**Table S1**: Estimated values from phi-coefficients (between binary variables), point-biserial correlations (between a binary and a continuous variable), and Spearman correlations (between continuous variables) among adversity variables experienced at birth.

|  | Impending maternal death | Primiparity | Hurricane environment | Population  density | Maternal age |
| --- | --- | --- | --- | --- | --- |
| Male infant |  |  |  |  |  |
| Impending maternal death | 1 |  |  |  |  |
| Primiparity | -0.030 | 1 |  |  |  |
| Hurricane environment | -0.006 | -0.022 | 1 |  |  |
| Population density | 0.028 | -0.134 | 0.198 | 1 |  |
| Maternal age | 0.234 | -0.731 | 0.014 | 0.083 | 1 |
| Female infant |  |  |  |  |  |
| Impending maternal death | 1 |  |  |  |  |
| Primiparity | -0.013 | 1 |  |  |  |
| Hurricane environment | -0.015 | -0.006 | 1 |  |  |
| Population density | -0.030 | -0.139 | 0.222 | 1 |  |
| Maternal age | 0.305 | -0.751 | -0.037 | 0.099 | 1 |

**Table S2**: Estimated values from phi-coefficients (between binary variables), point-biserial correlations (between a binary and a continuous variable), and Spearman correlations (between continuous variables) among adversity variables early in life.

|  | Competing sibling | Maternal loss | Primiparous | Hurricane | Population  density |
| --- | --- | --- | --- | --- | --- |
| Male adult |  |  |  |  |  |
| Competing sibling | 1 |  |  |  |  |
| Maternal loss | -0.152 | 1 |  |  |  |
| Primiparity | -0.240 | -0.041 | 1 |  |  |
| Hurricane | 0.009 | -0.058 | -0.032 | 1 |  |
| Population density | -0.030 | -0.010 | -0.154 | 0.457 | 1 |
| Female adult |  |  |  |  |  |
| Competing sibling | 1 |  |  |  |  |
| Maternal loss | -0.153 | 1 |  |  |  |
| Primiparity | -0.215 | -0.066 | 1 |  |  |
| Hurricane | -0.041 | -0.043 | -0.053 | 1 |  |
| Population density | -0.069 | -0.0004 | -0.152 | 0.432 | 1 |

**Table S3**. Hazard ratio estimated from Cox regression models testing associations between adversity at birth and rhesus macaque infant survival while controlling for maternal age.

|  | Hazard ratio | SE | 95% CI |
| --- | --- | --- | --- |
| *Males* (*n*=4,435) |  |  |  |
| *Individual effects* |  |  |  |
| Impending maternal death | **6.812** | **0.124** | **5.347, 8.678** |
| Maternal age | **0.833** | **0.041** | **0.769, 0.902** |
| Maternal age^2^ | **1.009** | **0.002** | **1.006, 1.013** |
| Hurricane environment | 0.927 | 0.195 | 0.633, 1.359 |
| Population density ≤ 2.28 months | 1.001 | 0.001 | 1.000, 1.003 |
| Population density > 2.28 months | **1.003** | **0.001** | **1.000, 1.005** |
| *Females* (*n*=4,195) |  |  |  |
| *Individual effects* |  |  |  |
| Impending maternal death | **5.783** | **0.131** | **4.470, 7.481** |
| Maternal age | **0.822** | **0.044** | **0.753, 0.896** |
| Maternal age^2^ | **1.009** | **0.002** | **1.005, 1.013** |
| Hurricane environment | 1.275 | 0.187 | 0.883, 1.841 |
| Population density ≤ 0.84 month | 0.999 | 0.001 | 0.997, 1.001 |
| Population density > 0.84 month | **1.006** | **0.001** | **1.003, 1.008** |

**Note**: Bold 95% CI indicate significance at 0.05 level (i.e., do not overlap with one).

**S4. Cox proportional hazards model with time-varying covariates for male rhesus macaques**

To extend our Cox models to time-varying covariates, we followed Zhang et al. (2018). Such method has been successfully applied to Cayo Santiago survival data in prior analyses (Lee et al. 2020). For parsimony, we tested the proportional hazards assumption following each stratification and kept the model with the least number of time cuts that satisfied the assumption.

Lee, D. S., Mandalaywala, T. M., Dubuc, C., Widdig, A., & Highams, J. P. (2020). Higher early life

mortality with lower infant body mass in a free-ranging primate. Journal of Animal Ecology, 89, 2300-2310.

Zhang, Z., Reinikainen, J., Adeleke, K. A., Pieterse, M. E., & Groothuis-Oudshoorn, C. G. M. (2018).

Time-varying covariates and coefficients in Cox regression models. *Annals of Translational Medicine*, *6*(7), 121-121.

**Adversity at birth and male infant survival.** When the proportional hazards (P-H) assumption was tested using a statistical test based on the scaled Schoenfeld residuals, the initial hazards model showed that the density and primiparity covariates violated the assumption. This indicated that the hazard of death associated to these two covariates was not constant over time:

|  | Chi-Squared | df | p-value |
| --- | --- | --- | --- |
| Impending maternal death | 2.760 | 1 | 0.10 |
| **Primiparity** | 4.298 | 1 | **0.04** |
| Hurricane environment | 0.122 | 1 | 0.73 |
| **Population density** | 4.677 | 1 | **0.03** |
| Global | 11.837 | 4 | **0.02** |


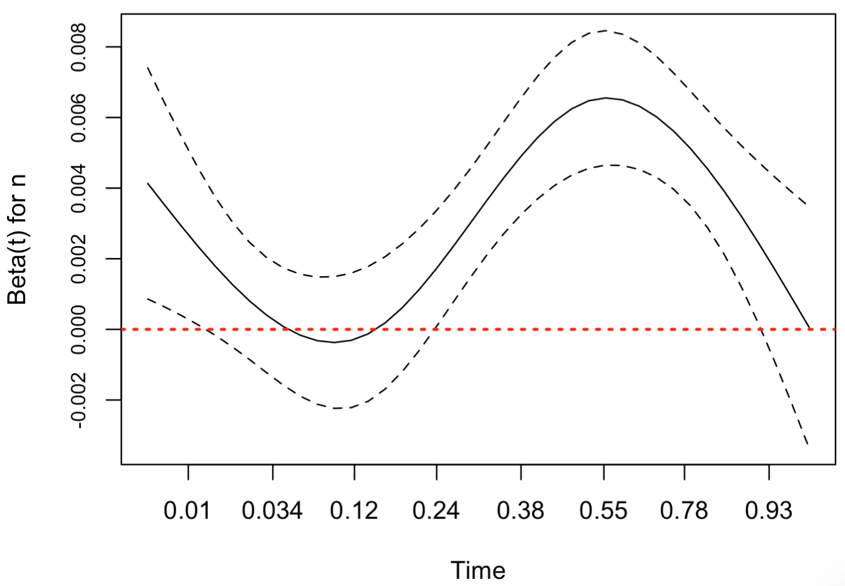


**Fig S4.1.** The hazard of death associated to population density (n) varies over time (age in years). The black dashed lines are lower and upper limits of confidence intervals of the effect of density and the red dashed line represents the reference line for the null effect.

Based on the visual inspection of Fig S4.1, the hazard of death is higher (i.e., positive beta estimate) for infants experiencing increased density until approximately the first 0.19 years, or 2.3 months, of age (the point where the slope of the beta reverses and exceeds the reference for null effect). Based on this observation, we postulated that there were two different age periods for the hazard of death related to density. To address this, we stratified density into two age periods (birth to 0.19 years and >0.19 years). After applying this method, the resulting hazards model satisfied the P-H assumption, as shown in the table below. Note that the same stratification was done for the analysis using maternal age (Table S3).

|  | Chi-Squared | df | p-value |
| --- | --- | --- | --- |
| Maternal death | 2.861 | 1 | 0.09 |
| Primiparity | 3.526 | 1 | 0.06 |
| Hurricane environment | 0.227 | 1 | 0.63 |
| Density | 0.050 | 1 | 0.82 |
| Density:strata | 0.312 | 1 | 0.58 |
| Global | 7.815 | 5 | 0.17 |

**Cumulative adversity at birth and male infant survival.** When the proportional hazards (P-H) assumption was tested using a statistical test based on the scaled Schoenfeld residuals, the initial hazards model showed that the cumulative adversity index violated the assumption. This indicated that the hazard of death associated to this covariate was not constant over time:

|  | Chi-Squared | df | p-value |
| --- | --- | --- | --- |
| Cumulative adversity index | 4.16 | 1 | **0.041** |
| Global | 4.16 | 1 | **0.041** |


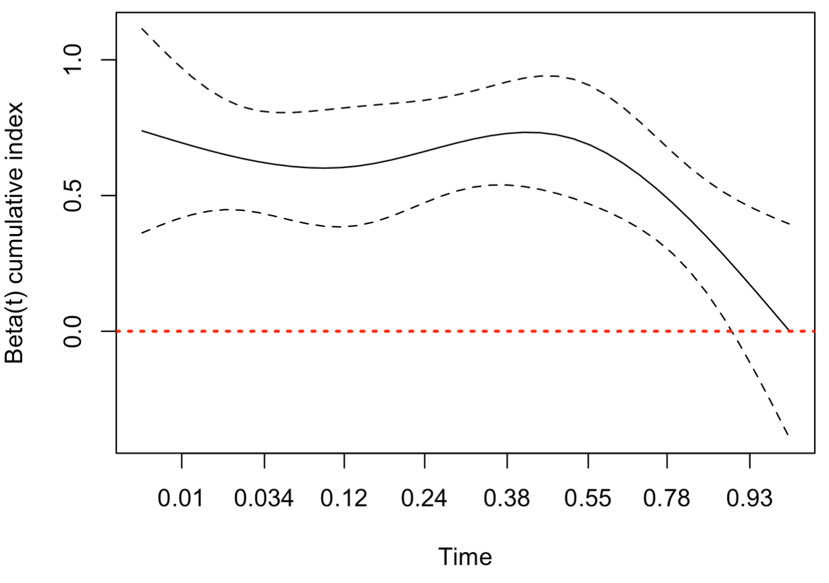


**Fig S4.2.** The hazard of death associated to cumulative adversity index varies over time (age in years). The black dashed lines are lower and upper limits of confidence interval of the effect of cumulative adversity and the red dashed line represents the reference line for the null effect.

Based on the visual inspection of Fig S4.2, the hazard of death is higher (i.e., positive beta estimate) for infants experiencing increased cumulative adversity at birth until approximately 0.90 years, or 10.8 months, of age (the point where the beta approaches 0 and the lower interval intercepts the reference for null effect). Based on this observation, we postulated that there were two different age periods for the hazard of death related to density. To address this, we stratified density into two age periods (birth to 0.90 years and >0.90 years). After applying this method, the resulting hazards model satisfied the P-H assumption, as shown in the table below.

|  | Chi-Squared | df | p-value |
| --- | --- | --- | --- |
| Cumulative adversity index | 0.335 | 1 | 0.56 |
| Cumulative adversity index:strata | 0.010 | 1 | 0.92 |
| Global | 0.341 | 2 | 0.84 |

**Early life adversity and male adult survival.** When the proportional hazards (P-H) assumption was tested using a statistical test based on the scaled Schoenfeld residuals, the initial hazards model showed that the hurricane and density covariates violated the P-H assumption. This indicated that the hazard of death associated to these covariates was not constant over time:

|  | Chi-Squared | df | p-value |
| --- | --- | --- | --- |
| Consecutive younger sibling | 1.252 | 1 | 0.26 |
| Maternal loss | 2.127 | 1 | 0.15 |
| Primiparity | 0.007 | 1 | 0.93 |
| **Hurricane** | 11.234 | 1 | **0.001** |
| **Density** | 5.484 | 1 | **0.02** |
| **Global** | 17.913 | 5 | **0.003** |


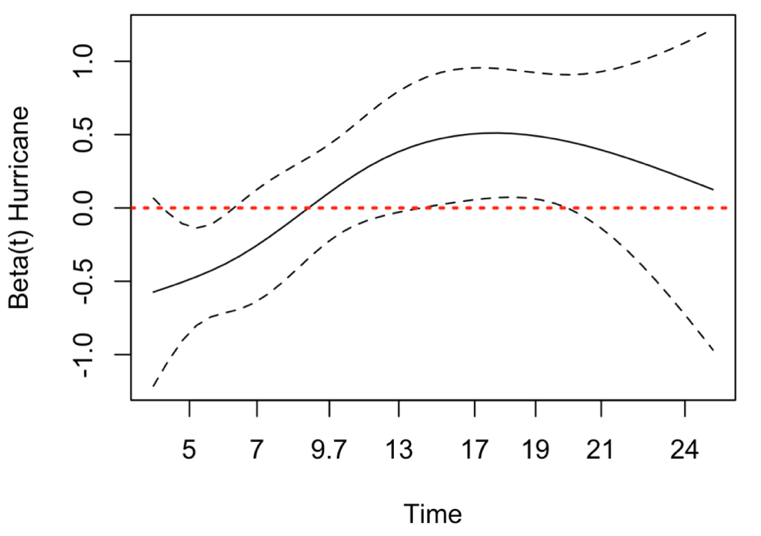


**Fig S4.3.** The hazard of death associated to hurricanes varies over time (age in years). The black dashed lines are lower and upper limits of confidence intervals of the effect of hurricanes and the red dashed line represents the reference line for the null effect.

Based on the visual inspection of Fig S4.3, the hazard of death is lower (i.e., negative beta estimate) for male adults experiencing a hurricane until approximately 8 years of age (the point where the beta exceeds the reference for null effect). Based on this observation, we postulated that there were two different age periods for the hazard of death related to hurricanes. To address this, we stratified the hurricane covariate into two age periods (3 to 8 years and >8 years). After applying this method, the resulting hazards model satisfied the P-H assumption, as shown in the table below.

|  | Chi-Squared | df | p-value |
| --- | --- | --- | --- |
| Consecutive younger sibling | 1.201 | 1 | 0.27 |
| Maternal loss | 1.989 | 1 | 0.16 |
| Primiparity | 0.026 | 1 | 0.87 |
| Hurricane | 0.441 | 1 | 0.51 |
| Hurricane:strata | 0.290 | 1 | 0.60 |
| Density | 3.565 | 1 | 0.06 |
| Global | 7.155 | 6 | 0.31 |

**Cumulative early life adversity and male adult survival.** When the proportional hazards (P-H) assumption was tested using a statistical test based on the scaled Schoenfeld residuals, the initial hazards model showed that the cumulative adversity index covariate violated the assumption. This indicated that the hazard of death associated to this covariate was not constant over time:

|  | Chi-Squared | df | p-value |
| --- | --- | --- | --- |
| Cumulative adversity | 5.96 | 1 | **0.015** |
| Global | 5.96 | 1 | **0.015** |


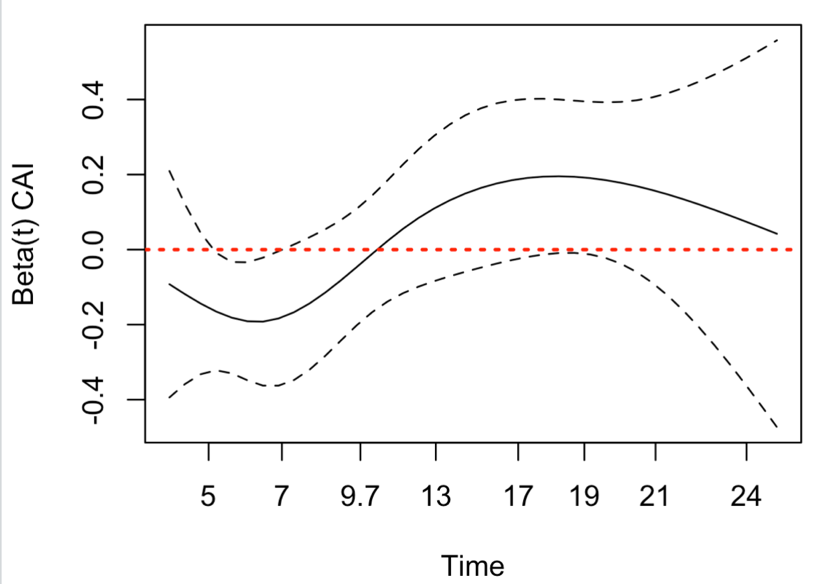


**Fig S4.4.** The hazard of death associated to the cumulative adversity index (CAI) covariate varies over time (age in years). The black dashed lines are lower and upper limits of confidence intervals of the effect of cumulative adversity and the red dashed line represents the reference line for the null effect.

Based on the visual inspection of Fig S4.4, the hazard of death is lower (i.e., negative beta estimate) for male adults experiencing increased cumulative adversity until approximately 10 years of age (the point where the beta exceeds the reference for null effect). Based on this observation, we postulated that there were two different age periods for the hazard of death related to cumulative adversity. To address this, we stratified the cumulative adversity index covariate into two age periods (3 to 10 years and >10 years). After applying this method, the resulting hazards model satisfied the P-H assumption, as shown in the table below.

|  | Chi-Squared | df | p-value |
| --- | --- | --- | --- |
| Cumulative adversity index | 0.012 | 1 | 0.91 |
| Cumulative adversity index:strata | 0.048 | 1 | 0.83 |
| Global | 0.063 | 2 | 0.97 |

**S5.** **Cox proportional hazards model with time-varying covariates for female rhesus macaques**

To extend our Cox models to time-varying covariates, we followed Zhang et al. (2018). Such method has been successfully applied to Cayo Santiago survival data in prior analyses (Lee et al. 2020). For parsimony, we tested the proportional hazards assumption following each stratification and kept the model with the least number of time cuts that satisfied the assumption.

Lee, D. S., Mandalaywala, T. M., Dubuc, C., Widdig, A., & Highams, J. P. (2020). Higher early life

mortality with lower infant body mass in a free-ranging primate. Journal of Animal Ecology, 89, 2300-2310.

Zhang, Z., Reinikainen, J., Adeleke, K. A., Pieterse, M. E., & Groothuis-Oudshoorn, C. G. M. (2018).

Time-varying covariates and coefficients in Cox regression models. *Annals of Translational Medicine*, *6*(7), 121-121.

**Adversity at birth and female infant survival.** When the proportional hazards (P-H) assumption was tested using a statistical test based on the scaled Schoenfeld residuals, the initial hazards model showed that the primiparity and density covariates violated the assumption. This indicated that the hazard of death associated to these covariates was not constant over time:

|  | Chi-Squared | df | p-value |
| --- | --- | --- | --- |
| Maternal death | 1.567 | 1 | 0.211 |
| **Primiparity** | 10.504 | 1 | **0.001** |
| Hurricane environment | 0.745 | 1 | 0.388 |
| **Density** | 18.664 | 1 | **<0.01** |
| **Global** | 30.772 | 4 | **<0.01** |


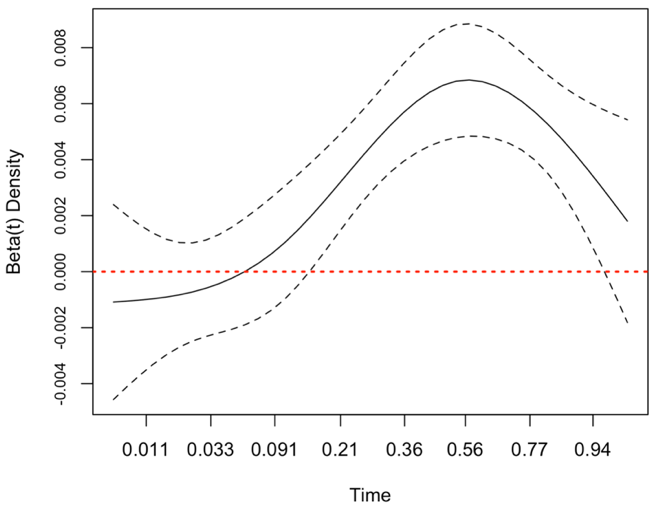


**Fig S5.1.** The hazard of death associated to the density covariate varies over time (age in years). The black dashed lines are lower and upper limits of confidence intervals of the effect of density and the red dashed line represents the reference line for the null effect.

Based on the visual inspection of Fig S5.1, the hazard of death is lower (i.e., negative beta estimate) for female infants experiencing increased density until approximately 0.07 years, or 0.84 months, of age (the point where the beta exceeds the reference for null effect). Based on this observation, we postulated that there were two different age periods for the hazard of death related to density. To address this, we stratified the density covariate into two age periods (birth to 0.07 years and >0.07 years). After applying this method, the primiparity covariate violated the P-H assumption:

|  | Chi-Squared | df | p-value |
| --- | --- | --- | --- |
| Maternal death | 1.356 | 1 | 0.24 |
| **Primiparity** | 8.729 | 1 | **0.003** |
| Hurricane environment | 1.644 | 1 | 0.20 |
| Density | 0.807 | 1 | 0.37 |
| Density:strata | 1.040 | 1 | 0.31 |
| **Global** | 13.103 | 5 | **0.02** |


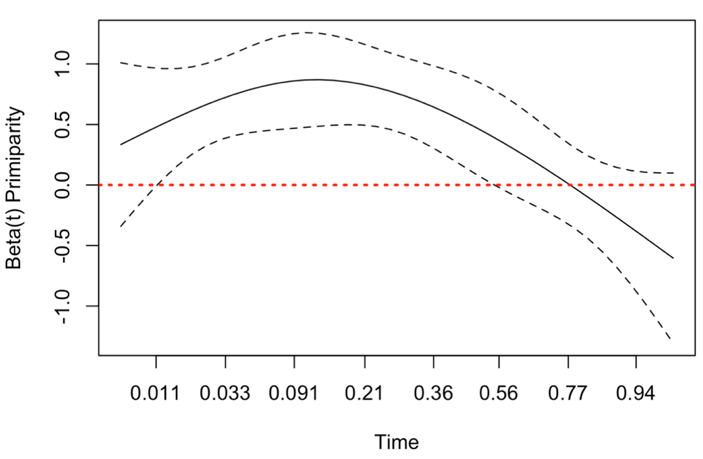


**Fig S5.2.** The hazard of death associated to the primiparity covariate varies over time (age in years). The black dashed lines are lower and upper limits of confidence intervals of the effect of primiparity, and the red dashed line represents the reference line for the null effect.

Based on the visual inspection of Fig S5.2, the hazard of death is higher (i.e., positive beta estimate) for female infants experiencing a primiparous mother until approximately 0.77 years, or 9.24 months, of age (the point where the beta intercepts the reference for null effect). Based on this observation, we postulated that there were two different age periods for the hazard of death related to primiparity. To address this, we stratified the primiparity covariate into two age periods (birth to 0.77 years and >0.77 years). After applying this method, the resulting hazards model satisfied the P-H assumption, as shown in the table below. Note that the same density stratification was done for the analysis using maternal age (Table S3).

|  | Chi-Squared | df | p-value |
| --- | --- | --- | --- |
| Maternal death | 1.739 | 1 | 0.19 |
| Primiparity | 1.384 | 1 | 0.24 |
| Primiparity:strata | 0.751 | 1 | 0.24 |
| Hurricane environment | 1.877 | 1 | 0.17 |
| Density | 0.763 | 1 | 0.24 |
| Density:strata | 0.859 | 1 | 0.35 |
| Global | 6.750 | 6 | 0.34 |

**Cumulative adversity at birth and female infant survival.** When the proportional hazards (P-H) assumption was tested using a statistical test based on the scaled Schoenfeld residuals, the initial hazards model showed that the cumulative adversity index covariate violated the P-H assumption. This indicated that the hazard of death related to cumulative adversity was not constant over time:

|  | Chi-Squared | df | p-value |
| --- | --- | --- | --- |
| Cumulative adversity index | 4.98 | 1 | **0.026** |
| Global | 4.98 | 1 | **0.026** |


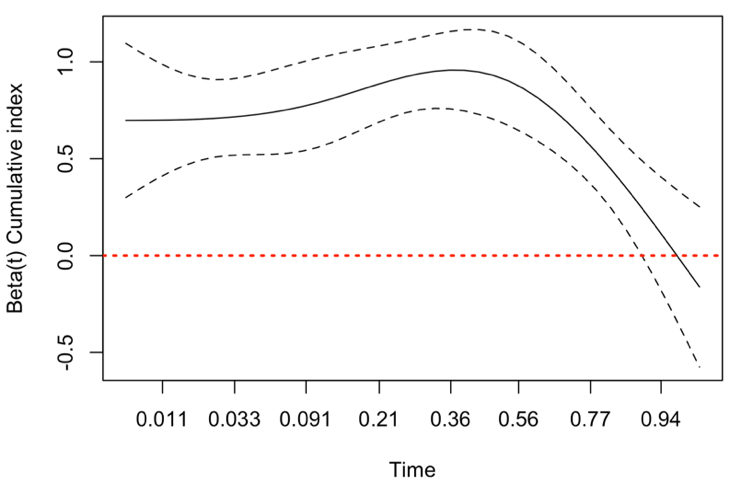


**Fig S5.3.** The hazard of death associated to the cumulative adversity index covariate varies over time (age in years). The black dashed lines are lower and upper limits of confidence intervals of the effect of cumulative adversity and the red dashed line represents the reference line for the null effect.

Based on the visual inspection of Fig S5.3, the hazard of death is higher (i.e., positive beta estimate) for female infants experiencing increased cumulative adversity at birth until approximately 0.90 years, or 10.8 months, of age (the point where the beta approaches 0 and the lower interval intercepts the reference for null effect). Based on this observation, we postulated that there were two different age periods for the hazard of death related to density. To address this, we stratified the density covariate into two age periods (birth to 0.90 years and >0.90 years). After applying this method, the resulting hazards model satisfied the P-H assumption, as shown in the table below.

|  | Chi-Squared | df | p-value |
| --- | --- | --- | --- |
| Cumulative adversity index | 0.105 | 1 | 0.75 |
| Cumulative adversity index:strata | 1.630 | 1 | 0.20 |
| Global | 1.713 | 2 | 0.42 |

**Early life adversity and female adult survival.** When the proportional hazards (P-H) assumption was tested using a statistical test based on the scaled Schoenfeld residuals, the initial hazards model showed that the maternal loss and density covariates violated the P-H assumption. This indicated that the hazard of death related to these two covariates was not constant over time:

|  | Chi-Squared | df | p-value |
| --- | --- | --- | --- |
| Consecutive younger sibling | 0.244 | 1 | 0.621 |
| **Maternal loss** | 0.113 | 1 | **0.001** |
| Primiparity | 8.570e-3 | 1 | 0.926 |
| Major hurricane | 1.520e-5 | 1 | 0.997 |
| **Density** | 5.890 | 1 | **0.015** |
| **Global** | 14.700 | 5 | **0.012** |


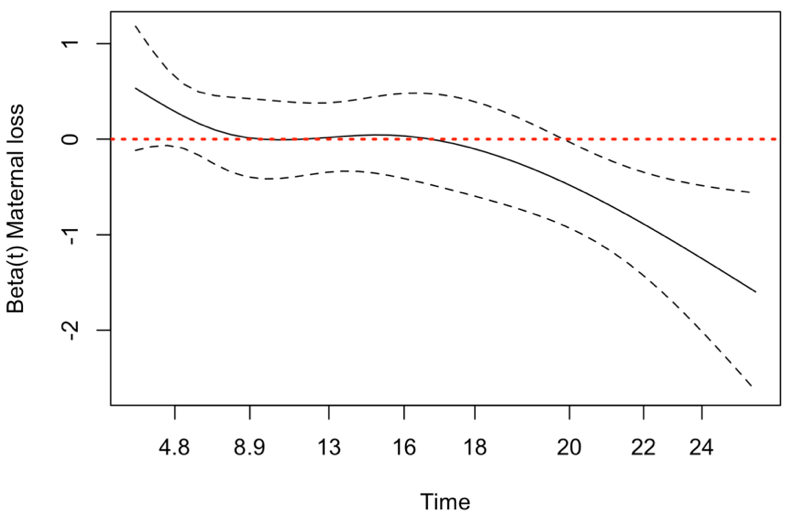


**Fig S5.4.** The hazard of death associated to the maternal loss covariate varies over time (age in years). The black dashed lines are lower and upper limits of confidence interval of the effect of maternal loss and the red dashed line represents the reference line for the null effect.

Based on the visual inspection of Fig S5.4, the hazard of death diminishes (i.e., approaches to and overlaps with 0) for female adults experiencing maternal loss early in life until approximately 18 years of age (the point after the beta intercepts the reference for null effect and the slope becomes to decrease at higher rates). Based on this observation, we postulated that there were two different age periods for the hazard of death related to density. To address this, we stratified the density covariate into two age periods (3 to 18 years and >18 years). After applying this method, the resulting hazards model satisfied the P-H assumption, as shown in the table below.

|  | Chi-Squared | df | p-value |
| --- | --- | --- | --- |
| Consecutive younger sibling | 0.335 | 1 | 0.56 |
| Maternal loss | 0.729 | 1 | 0.39 |
| Maternal loss:strata | 0.024 | 1 | 0.88 |
| Primiparity | 0.046 | 1 | 0.83 |
| Major hurricane | 0.087 | 1 | 0.77 |
| Density | 2.375 | 1 | 0.12 |
| Global | 3.907 | 6 | 0.69 |
